# FlyDEGdb knowledge base on differentially expressed genes of Drosophila melanogaster, a model object in biomedicine

**DOI:** 10.18699/vjgb-25-101

**Published:** 2025-12

**Authors:** O.A. Podkolodnaya, M.A. Deryuzhenko, N.N. Tverdokhleb, K.A. Zolotareva, Yu.V. Makovka, N.L. Podkolodny, V.V. Suslov, I.V. Chadaeva, L.A. Fedoseeva, A.A. Seryapina, D.Yu. Oshchepkov, A.G. Bogomolov, E.Yu. Kondratyuk, O.E. Redina, A.L. Markel, N.E. Gruntenko, M.P. Ponomarenko

**Affiliations:** Institute of Cytology and Genetics of the Siberian Branch of the Russian Academy of Sciences, Novosibirsk, Russia; Institute of Cytology and Genetics of the Siberian Branch of the Russian Academy of Sciences, Novosibirsk, Russia; Institute of Cytology and Genetics of the Siberian Branch of the Russian Academy of Sciences, Novosibirsk, Russia; Institute of Cytology and Genetics of the Siberian Branch of the Russian Academy of Sciences, Novosibirsk, Russia; Institute of Cytology and Genetics of the Siberian Branch of the Russian Academy of Sciences, Novosibirsk, Russia; Institute of Cytology and Genetics of the Siberian Branch of the Russian Academy of Sciences, Novosibirsk, Russia Institute of Computational Mathematics and Mathematical Geophysics of the Siberian Branch of the Russian Academy of Sciences, Novosibirsk, Russia; Institute of Cytology and Genetics of the Siberian Branch of the Russian Academy of Sciences, Novosibirsk, Russia; Institute of Cytology and Genetics of the Siberian Branch of the Russian Academy of Sciences, Novosibirsk, Russia; Institute of Cytology and Genetics of the Siberian Branch of the Russian Academy of Sciences, Novosibirsk, Russia; Institute of Cytology and Genetics of the Siberian Branch of the Russian Academy of Sciences, Novosibirsk, Russia; Institute of Cytology and Genetics of the Siberian Branch of the Russian Academy of Sciences, Novosibirsk, Russia; Institute of Cytology and Genetics of the Siberian Branch of the Russian Academy of Sciences, Novosibirsk, Russia; Institute of Cytology and Genetics of the Siberian Branch of the Russian Academy of Sciences, Novosibirsk, Russia Siberian Federal Scientific Centre of Agro-BioTechnologies of the Russian Academy of Sciences, Krasnoobsk, Novosibirsk region, Russia; Institute of Cytology and Genetics of the Siberian Branch of the Russian Academy of Sciences, Novosibirsk, Russia; Institute of Cytology and Genetics of the Siberian Branch of the Russian Academy of Sciences, Novosibirsk, Russia Novosibirsk State University, Novosibirsk, Russia; Institute of Cytology and Genetics of the Siberian Branch of the Russian Academy of Sciences, Novosibirsk, Russia; Institute of Cytology and Genetics of the Siberian Branch of the Russian Academy of Sciences, Novosibirsk, Russia

**Keywords:** human, disease, biomedicine, model animal, fruit fly Drosophila melanogaster, differentially expressed genes (DEGs), RNA-Seq, qPCR, microarray, knowledge base, человек, заболевание, биомедицина, модельное животное, дрозофила, Drosophila melanogaster, дифференциально экспрессирующиеся гены (ДЭГ), RNA-seq, qPCR, микрочип, база знаний

## Abstract

Since the work of Nobel Prize winner Thomas Morgan in 1909, the fruit fly Drosophila melanogaster has been one of the most popular model animals in genetics. Research using this fly was honored with the Nobel Prize many times: in 1946 (Muller, X-ray mutagenesis), in 1995 (Lewis, Nüsslein-Volhard, Wieschaus, genetic control of embryogenesis), in 2004 (Axel and Buck, the olfactory system), in 2011 (Steinman, dendritic cells in adaptive immunity; Beutler and Hoffman, activation of innate immunity), and in 2017 (Hall, Rosbash and Young, the molecular mechanism of the circadian rhythm). The prominent role of Drosophila in genetics is due to its key features: short life cycle, frequent generational turnover, ease of maintenance, high fertility, small size, transparent embryos, simple larval structure, the possibility to observe visually chromosomal rearrangements due to the presence of polytene chromosomes, and accessibility to molecular genetic manipulation. Furthermore, the highly conserved nature of several signaling pathways and gene networks in Drosophila and their similarity to those of mammals and humans, taken together with the development of high-throughput genomic sequencing, motivated the use of D. melanogaster as a model organism in biomedical fields of inquiry: pharmacology, toxicology, cardiology, oncology, immunology, gerontology, and radiobiology. These studies add to the understanding of the genetic and epigenetic basis of the pathogenesis of human diseases. This paper describes our curated knowledge base, FlyDEGdb (https://www.sysbio.ru/FlyDEGdb), which stores information on differentially expressed genes (DEGs) in Drosophila. This information was extracted from 50 scientific articles containing experimental data on changes in the expression of 20,058 genes (80 %) out of the 25,079 Drosophila genes stored in the NCBI Gene database. The changes were induced by 52 stress factors, including heat and cold exposure, dehydration, heavy metals, radiation, starvation, household chemicals, drugs, fertilizers, insecticides, pesticides, herbicides, and other toxicants. The FlyDEGdb knowledge base is illustrated using the example of the dysf (dysfusion) Drosophila gene, which had been identified as a DEG under cold shock and in toxicity tests of the herbicide paraquat, the solvent toluene, the drug menadione, and the food additive E923. FlyDEGdb stores information on changes in the expression of the dysf gene and its homologues: (a) the Clk, cyc, and per genes in Drosophila, and (b) the NPAS4, CLOCK, BMAL1, PER1, and PER2 genes in humans. These data are supplemented with information on the biological processes in which these genes are involved: oocyte maturation (oogenesis), regulation of stress response and circadian rhythm, carcinogenesis, aging, etc. Therefore, FlyDEGdb, containing information on the widely used model organism, Drosophila, can be helpful for researchers working in the molecular biology and genetics of humans and animals, physiology, translational medicine, pharmacology, dietetics, agricultural chemistry, radiobiology, toxicology, and bioinformatics.

## Abstract (ru)

С 1909 г. благодаря исследованиям нобелевского лауреата Моргана дрозофила Drosophila melanogaster стала одним из самых популярных модельных животных в генетике. Фундаментальные исследования с дрозофилой в качестве модельного объекта неоднократно были отмечены Нобелевской премией: в 1946 г. (Мёллер, мутагенез при рентгеновском излучении), в 1995 (Льюис, Нюссляйн-Фольхард, Вишаус, генетический контроль эмбриогенеза), в 2004 (Эксел и Бак, обонятельная система), в 2011 (Стайнман, дендритные клетки в адаптивном иммунитете; Бётлер и Офман, активация врожденного иммунитета) и в 2017 г. (Холл, Росбаш и Янг, молекулярный механизм циркадного ритма). Столь яркая роль дрозофилы в генетике обусловлена рядом ее ключевых признаков: кратким жизненным циклом, частой сменой поколений, легкостью в содержании, высокой плодовитостью, малым размером, прозрачностью эмбриона, простым строением личинки, возможностью визуальных наблюдений хромосомных перестроек за счет наличия политенных хромосом, доступностью для молекулярно-генетических манипуляций. Кроме того, благодаря высокой консервативности ряда сигнальных путей и генных сетей дрозофилы и их сходству с таковыми у млекопитающих и человека в совокупности с техническим развитием геномного секвенирования стало возможно использование D. melanogaster как модельного объекта в биомедицинских исследованиях в области фармакологии, токсикологии, кардиологии, онкологии, иммунологии, геронтологии и радиобиологии для поиска генетической и эпигенетической основ патогенеза болезней человека. В настоящей статье описана созданная нами курируемая база знаний FlyDEGdb (https://www.sysbio.ru/FlyDEGdb), в которой представлена информация о дифференциально экспрессирующихся генах (ДЭГ) дрозофилы, экстрагированная из 50 научных статей с экспериментальными данными об изменении экспрессии 20 058 генов (80 %) из числа всех 25 079 генов дрозофилы согласно базе данных NCBI Gene под действием 52 стрессовых факторов, включая высокую и низкую температуры, обезвоживание, тяжелые металлы, радиацию, голод, яды, бытовую химию, лекарства, удобрения, инсектициды, пестициды и гербициды. Содержание базы знаний FlyDEGdb проиллюстрировано на примере гена dysf (dysfusion) дрозофилы, который был идентифицирован в качестве ДЭГ при множестве стрессовых воздействий: холодовом шоке и в испытаниях на токсичность гербицида параквата, растворителя толуола, лекарственного препарата менадиона, пищевой добавки Е923. В FlyDEGdb представлена информация об изменениях экспрессии гена dysf и его гомологов Clk, cyc, per у дрозофилы и генов NPAS4, CLOCK, BMAL1, PER1 и PER2 человека, а также информация о биологических процессах, в которые вовлечены эти гены: созревание ооцитов (оогенез), регуляция стресс-ответа и циркадного ритма, канцерогенез, старение и др. Поэтому FlyDEGdb, содержащая информацию о таком модельном организме, как дрозофила, может быть полезна для исследователей, работающих в области молекулярной биологии и генетики человека и животных, физиологии, трансляционной медицины, фармакологии, диетологии, агрохимии, радиобиологии, токсикологии и биоинформатики.

## Introduction

Animal models are broadly employed in biomedical studies of the physiological, genetic, and epigenetic mechanisms regulating evolutionarily fixed phenotypic human traits in health and disease, as well as in response to external and internal stress factors (Mukherjee et al., 2022). Their use is based on strict criteria of the correspondence between the human phenotypic features under study and their counterparts in model animals (Gryksa et al., 2023). Over a century ago, Thomas Hunt Morgan (1910), Professor of Experimental Zoology in the Columbia University, laid the foundation of a series of discoveries in heredity in a then new biological object, Drosophila melanogaster. His results were honored with the Nobel Prize “For his discoveries concerning the role played by the chromosome in heredity” in 1933. Later genetic studies using Drosophila were honored with the Nobel Prize five times more. In 1946, it was awarded to Hermann Muller “For the discovery of the production of mutations by means of X-ray irradiation”; in 1995, to Edward Lewis, Christiane Nüsslein- Volhard, and Eric Wieschaus “For their discoveries concern ing the genetic control of early embryonic development”; in 2004, to Richard Axel and Linda Buck “For their discoveries of odorant receptors and the organization of the olfactory system”; in 2011, to Ralph Steinman “For his discovery of the dendritic cell and its role in adaptive immunity” together with Jules Hoffman and Bruce Beutler “For their discoveries concerning the activation of innate immunity”; and in 2017, to Jeffrey Hall, Michael Rosbash, and Michael Young “For their discoveries of molecular mechanisms controlling the circadian rhythm” (Lakhotia, 2025).

This great significance of Drosophila for research is determined by the low cost of their maintenance, high fertility, frequent generation turnover, small size, optical transparency of embryos, simple larva structure, short life cycle, availability of numerous natural strains adapted to various ecoclimatic conditions (Telonis-Scott et al., 2013; Chen et al., 2015; von Heckel et al., 2016; Mikucki et al., 2024), relatively small genome, and ease of molecular genetic manipulations. It is of special importance that many signaling pathways and gene networks of Drosophila are similar to those of the human (Yu et al., 2022). Owing to this fact, many results in translational medicine, pharmacology, toxicology, immunology, gerontology, etc. obtained with Drosophila can be transferred to humans (De Gregorio et al., 2001; Chatterjee, Perrimon, 2021; Wu K. et al., 2021; Ali et al., 2022; Rand et al., 2023).

Within this line of inquiry, scientists of the Institute of Cytology and Genetics (ICG) of the Siberian Branch of the Russian Academy of Sciences, Novosibirsk, have investigated features of stress response in rats (Markel, 1985; Oshchepkov et al., 2024) and mice (Chadaeva et al., 2019; Avgustinovich et al., 2025) for over 40 years. The results, reported in many publications, present valuable data on changes in gene expression induced by various experimental procedures. Huge volumes of genome-wide data (Big Data) on DEGs in rats and mice have been obtained and documented in our knowledge bases RatDEGdb (Chadaeva et al., 2023) and MiceDEGdb (Podkolodnaya et al., 2024), respectively.

D. melanogaster is another model species, in which experiments on stress in animals have been conducted at ICG for over 25 years (Gruntenko et al., 1999; 2023). The effort on developing the FlyDEGdb knowledge base, which stores information on Drosophila DEGs, is the continuation of our works in biomedical knowledge bases RatDEGdb and MiceDEGdb. The pilot version of FlyDEGdb v.0.1 is freely available at https://www.sysbio.ru/FlyDEGdb. It stores experimental data on the expression of 80 % of Drosophila genes: 20,058 of the 25,079 annotated in NCBI Gene (Brown et al., 2015). The information presented in FlyDEGd was extracted from 50 papers reporting experimental data on the action of 52 stress factors on 31 D. melanogaster strains. The factors included heat and cold, dehydration, heavy metals, radiation, starvation, household chemicals, drugs, fertilizers, insecticides, pesticides, herbicides, and other toxicants. The informational content of FlyDEGdb v0.01 is illustrated by the Drosophila dysf (dysfusion) gene, which was identified as a DEG in cold shock and in tests of the herbicide paraquat, solvent toluene, drug menadione, and food additive E923. FlyDEGdb presents data on changes in the expression of dysf itself and its homologs: Clk, cyc, and per in Drosophila and NPAS4, CLOCK, BMAL1, PER1, and PER2 in the human. In addition, FlyDEGdb provides information on the biologic processes involving these genes: oogenesis, regulation of stress response and circadian rhythms, carcinogenesis, aging, etc.

We also compare data on stress-induced Drosophila DEGs presented in FlyDEGdb with data on changes in the expression of DEGs of the hypothalamus of rat strains WAG and ISIAH in response to restriction stress, reported by D.Y. Oshchepkov et al. (2024) and presented in RatDEGdb. The responses of rats and Drosophila to stresses reveal a common molecular event: reduction in the expression of large gene groups involved in the formation of the plasma membrane. The FlyDEGdb knowledge base, storing information on the model species Drosophila, can be a useful tool for students of the molecular biology and genetics of the human and animals, physiology, translational medicine, pharmacology, nutrition science, agricultural chemistry, radiobiology, toxicology, and bioinformatics

## Materials and methods

**Stress-inducible Drosophila DEGs.** Experimentally detected Drosophila DEGs were sought in the PubMed database (Lu, 2011) with queries composed from various combinations of key words “Drosophila melanogaster”, “differentially expressed gene”, “stress response”, “drying”, “heat shock”, “radiation”, “cold shock”, “oxidative stress”, “continuous lighting”, “toxin”, “diet”, “heavy metal”, “drug”, “herbicide”, “pesticide”, “insecticide”, “RNA-seq”, “microarray”, and “qPCR”.

Only DEGs with reported log2(DEG) = log2([DEG expression in Drosophila under a particular stress factor] / [normal DEG expression]) values and PADJ estimates of statistical significance with correction for multiple comparisons for the stress-induced expression of the DEG were added to FlyDEGdb. In addition, we eliminated those in which the log2(DEG) values ranged from –0.46 to 0.46. This range corresponds to statistically insignificant ( p ≥ 0.05, Fisher’s Z-test) differences in DEG expression before and after the exposure to stress with ±5 % accuracy of expression measurements.

**Fig. 1.**
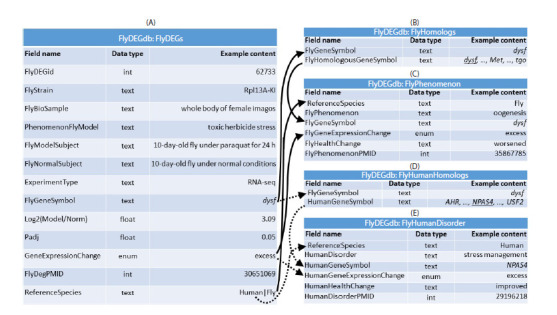
The informational structure of the FlyDEGdb knowledge base on differentially expressed genes (DEGs) of Drosophila melanogaster. Relational tables: (A) FlyDEGs – experimental data on DEGs in Drosophila tissue samples in response to a stress factor relative to the norm according to the paper cited; (B) FlyHomologs – lists of Drosophila genes homologous to particular Drosophila genes according to the FlyBase database (Ozturk-Colak et al., 2024); (C) FlyPhenomenon – phenotypic traits associated with deviations in the expression of Drosophila genes relative to the norm according to the paper cited; (D) FlyHumanHomologs – human genes homologous to a particular Drosophila gene according to FlyBase (Ozturk-Colak et al., 2024); (E) HumanDisorder – human diseases associated with deviations in particular human genes relative to the norm according to the paper cited. Names of relational tables and their fields were chosen following the guideline on the construction of friendly interfaces (Wade, 1984). Data types: int – integer number; float – real number; enum – binary indicator; text – character string; PMID – identifier of the referred paper in PubMed (Lu, 2011). Arrows (→) – relational links pointing to the annotation of experimental data on Drosophila DEGs (relational table FlyDEGs) on the one side and, on the other side, data on ipsidirectional changes in the expression of homologous Drosophila (solid lines) or human (dotted lines) DEGs indicated in the FlyHomologs and FlyHumanHomologs tables, obtained in independent experiments referred to in relational tables FlyPhenomenon and HumanDisorder.

**FlyDEGdb knowledge base.**Figure 1 illustrates the informational structure of the FlyDEGdb knowledge base. It includes five relational tables. The first of them, named “FlyDEGs” (Fig. 1A), stores experimental data on a particular Drosophila DEG, which is assigned a unique number (field “FlyDEGid”). Field “FlyStrain” of the table indicates the Drosophila strain in which the DEG has been found in experiments. Field “FlyBioSample” indicates the tissue sample studied in the experiment. Field “PhenomenonFlyModel” indicates the corresponding stress factor. Fields “FlyModel Subject” and “FlyNormalSubject” indicate the model and control individuals, respectively, used in the experiment. The experiment type, “RNA-seq”, “Microarray”, or “RT-qPCR”, is shown in field “ExperimentType”. Field “FlyGeneSymbol” contains the identifier of the Drosophila DEG according to the NCBI Gene database (Brown et al., 2015). Fields “Log2(Model/Norm)” and “Padj” contain the quantity of the stress-induced change in DEG expression as compared to the norm and its significance level with correction for multiple comparisons, respectively, as they are reported. The source is indicated in field “FlyDegPMID” as its identifier in PubMed (Lu, 2011).

Finally, field “ReferenceSpecies” indicates the reference biologic species (“Fly” for Drosophila or “Human” for the human in the pilot version FlyDEGdb v0.1), the experimental data on which are used in the annotation of a particular DEG. Absence of such annotation is indicated as “ND”.

Here we apply the term “annotation” to the supplementation of experimental data on stress-induced changes in the expression of a particular Drosophila DEG reported in a particular paper with experimental data from independent sources on phenotypic manifestations of ipsidirectional changes in the expression of homologous human and Drosophila genes. Supplementary Table S11 provides details of the annotation procedure


Supplementary Materials are available in the online version of the paper: https://vavilov.elpub.ru/jour/manager/files/Suppl_Podkol_Engl_29_7.pdf


To conclude the description of the informational structure of FlyDEGdb (Fig. 1), we indicate the data types used: int, integer number; float, real number; enum, binary indicator; text, character string.

The relational tables FlyDEGs, FlyHomologs, FlyPhenomenon, FlyHumanHomologs, and HumanDisorder were integrated to the FlyDEGdb knowledge base (https://www. sysbio.ru/FlyDEGdb) by using the MySQL-compatible database management studio MariaDB 10.2.12 (MariaDB Corp AB, Finland).

**Statistical methods.** The statistical analysis of Drosophila DEGs was conducted with Past v.4.04 application (Hammer et al., 2001) and the STATISTICA package (Statsoft™, United States

## Results and discussion

**FlyDEGdb knowledge base**

We sought papers on Drosophila DEGs in PubMed (Lu, 2011) with keywords listed in section “Materials and methods” to populate FlyDEGdb. We found 51 articles describing 287 experiments on 31 Drosophila strains originating from various geographical areas and their transgenic modifications. The articles described over 190,000 stress-inducible Drosophila DEGs. The results of the search are shown in Tables S1–S3. The articles cover a wide range of Drosophila studies concerning age-related human diseases, Drosophila tests of drugs, and tests for toxicity of household chemicals, fertilizers, insecticides, pesticides, herbicides, etc.

**Fig. 2.**
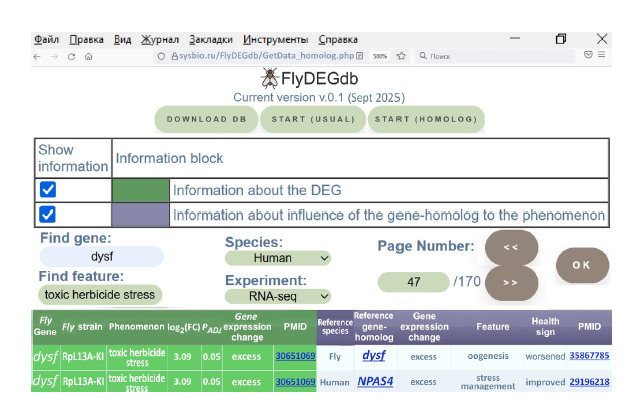
The interface of the FlyDEGdb knowledge base on D. melanogaster DEGs supports the real-time dialogue for user access to the informational content. Interface commands: DOWNLOAD DB – download the entire body of information of the current version FlyDEGdb v0.1 as a text file in an Excel-compatible format; START (HOMOLOG) – access to Drosophila DEGs annotated with the use of independent experimental data on the phenotypic manifestation of ipsidirectional expression changes relative to normal values in homologous genes in reference biologic species: Drosophila and the human; START (USUAL) – access to Drosophila DEGs omitting annotation. Left half of the table with information on Drosophila DEG (green background): experimental data on the Drosophila DEG considered; right half (lilac background): annotation of the DEG on the grounds of independent data on the phenotypic manifestation of ipsidirectional expression changes in homologous genes in reference biologic species: Drosophila and the human

Figure 2 illustrates user access to the information stored in the pilot FlyDEGdb version.

Three buttons at the top of the FlyDEGdb interface provide access to the information:

• “DOWNLOAD DB” allows downloading all information from the current version FlyDEGdb v0.1 as a text file in an Excel-compatible format. • “START (USUAL)” provides access to experimental data on stress-inducible Drosophila DEGs described in the main relational Table “FlyDEGs” (Fig. 1A). • “START (HOMOLOG)” provides access to the annotations of Drosophila DEGs as described in section “Materials and methods”.

Below there are interface fields for choosing the needed type of information: experimental data on Drosophila DEGs and/or annotation of Drosophila DEGs. The “Page Number” field allows alphabetical navigation over all DEGs stored in the knowledge base.

The bottom part of the interface outputs tabulated information on DEGs obtained by the user according to the specified query. Its description is provided in section “Materials and methods”. Their storage in FlyDEGdb is shown in Figure 1.

Table 1 provides a detailed description of the Drosophila dysf DEG in response to various stress factors, as well as information on homologous DEGs in Drosophila and the human. Seven columns on the left contain experimental data on Drosophila dysf in response to the toxic effect of the herbicide paraquat, which increases the expression, and toluene, which decreases it. The expression changes are characterized by log2(DEG) values and significance levels PADJ. Column PMID indicates information sources.

**Table 1.**
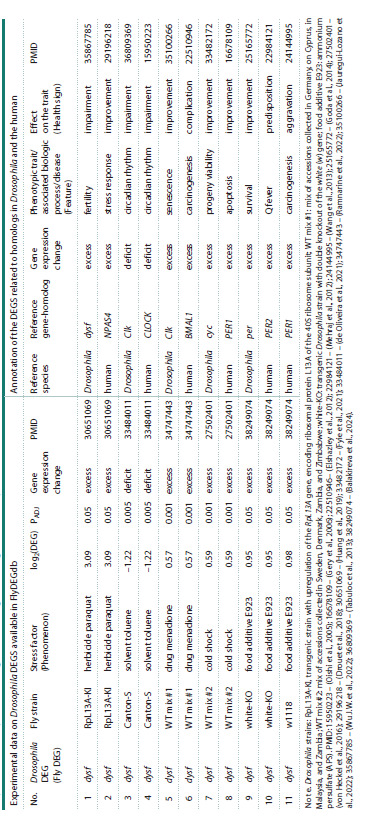
Examples of the presentation of experimental data on Drosophila DEGs in the FlyDEGdb knowledge base and their annotations related to data on the phenotypic manifestations of ipsidirectional expression changes of homologous DEGs genes in Drosophila and the human Notе. Drosophila strains: RpL13A-KI, transgenic strain with upregulation of the RpL13A gene, encoding ribosomal protein L13A of the 40S ribosome subunit; WT mix #1: mix of accessions collected in Germany, on Cyprus, in Malaysia, and Zambia; WT mix #2: mix of accessions collected in Sweden, Denmark, Zambia, and Zimbabwe; white-KO: transgenic Drosophila strain with double knockout of the white (w) gene; food additive E923: ammonium persulfate (APS). PMID: 15950223 – (Oishi et al., 2005); 16678109 – (Gery et al., 2006); 22510946 – (Elshazley et al., 2012); 22984121 – (Mehraj et al., 2012); 24144995 – (Wang et al., 2013); 25165772 – (Goda et al., 2014); 27502401 – (von Heckel et al., 2016); 29196218 – (Drouet et al., 2018); 30651069 – (Huang et al., 2019); 33482172 – (Fyie et al., 2021); 33484011 – (de Oliveira et al., 2021); 34747443 – (Ramnarine et al., 2022); 35100266 – (Jauregui-Lozano et al., 2022); 35867785 – (Wu J.W. et al., 2022); 36809369 – (Tabuloc et al., 2013); 38249074 – (Balakireva et al., 2024).

Six columns on the right in Table 1 contain the results of annotation of the Drosophila dysf gene compared to the homologous Clk gene of the same species and to homologous human genes NPAS4 and CLOCK on the base of four independent PMID papers. It is apparent that (a) the dysf upregulation (excess) is associated with Drosophila oogenesis impairments; (b) the downregulation (deficit) of the Clk gene, homologous to Drosophila dysf, disrupts the circadian rhythm; (c) the upregulation of the human NPAS4 gene, homologous to Drosophila dysf, improves the efficiency of the stress response; (d) the downregulation of the human CLOCK gene, homologous to Drosophila dysf, disrupts the circadian rhythm. Similar examples are shown in rows 5–11.

**Comparison of stress-induced homologous rat and Drosophila genes on the grounds of information from FlyDEGdb and RatDEGdb**

Table 2 presents information on DEGs detected in a restriction stress experiment in the hypothalamus or WAG and ISIAH rats (Oshchepkov et al., 2024) in comparison with homologous Drosophila DEGs described in FlyDEGdb

**Table 2.**
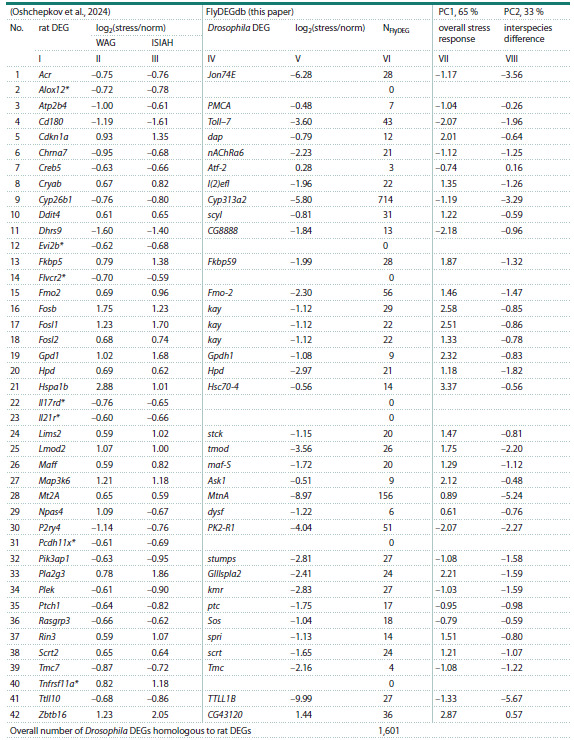
Using the FlyDEGdb knowledge base to the analysis of DEG expression in the hypothalamus of WAG and ISIAH rats in response to restriction stress (Oshchepkov et al., 2024) Notе. NFlyDEG – number of Drosophila DEGs homologous to the rat DEG according to FlyBase (Ozturk-Colak et al., 2024). * Rat genes (Flvcr2, Alox12, Evi2b, Il17rd, Il21r, Pcdh11x, Tnfrsf11a), for which no homologous Drosophila genes are found in FlyDEGdb v0.1 (NFlyDEG = 0).

Consider the representation of this information by the example of the first row of the table. It describes the rat Acr DEG. Column I indicates the gene name; columns II and III, stress-induces changes in its expression in rats of the WAG and ISIAH strains, respectively. Column IV indicates the name of the homologous Drosophila Jon74E gene; column V, the magnitude of its expression change; and column VI shows the total number of such Drosophila DEGs homologous to Acr.

Columns VII and VIII show the values of the first (PC1) and second (PC2) principal components revealed in the analysis of the above-described experimental data on the magnitude of stress-induced change in Drosophila DEG expression from FlyDEGdb and homologous rat genes from RatDEGdb (Oshchepkov et al., 2024). The analysis was conducted with Past v.4.04 software (Hammer et al., 2001).

The first principal component (PC1) is the weighted-mean estimate of the overall stress-induced change in the expression of homologous Drosophila (DEGFLY) and rat (DEGISIAH and DEGWAG) genes:

**Formula. 1.**
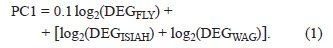
Formula 1

Principal component PC1 explains 65 % of the variance in the entire set of the considered experimental data on homologous rat and Drosophila DEGs.

Principal component PC2 is the weighted-mean estimate of the interspecies difference between Drosophila and rat in stress-induces changes in the expression of DEGs and their homologs:

**Formula. 2.**
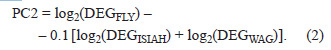
Formula 2

Principal component PC2 explains 33 % of the variance in the considered experimental data.

Thus, we were first to find that two-thirds (65 %) of the variance in gene expression change in the rat and Drosophila exposed to stress were determined by common mechanisms of response to stress (PC1), and one-third (33 %) reflects interspecies difference between the rat and Drosophila (PC2).

The statistical significance ( p < 0.05) of principal components PC1 and PC2 found in our study was deduced from 1,000 bootstrap samples with a special module of Past v.4.04 software (Hammer et al., 2001) (Fig. S1).

**Fig. 3.**
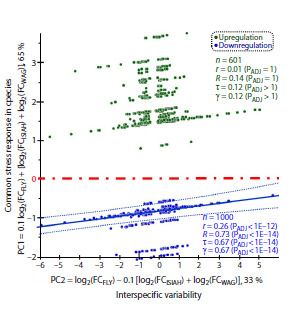
Results of the correlation analysis between principal components PC1 and PC2 for experimental data on pairs of DEGs homologous between the rat and Drosophila (Table 1). Principal components: PC1, Y axis; PC2, X axis. Each point corresponds to the values calculated for a certain pair of DEGs: Drosophila gene from FlyDEGdb and its homolog from RatDEGdb. The red dash-dotted line is the boundary between figure areas for stress-induced downregulation (blue) and upregulation (green) according to the PC1 estimate by Equation (1); the solid line reflects the linear correlation between PC1 and PC2 at PC1 < 0; the dotted lines border the 95 % confidence range for the correlation; alphabetical designations r, γ, R, τ, and PADJ are correlation coefficients, respectively: linear correlation, Goodman– Kruskal generalization; Spearman–Kendal rank correlation, and their statistical significance levels with Bonferroni correction for multiple comparisons, as calculated with Statistica software (Statsoft™, United States).

The numerical values of PC1 and PC2 are shown in columns VII and VIII of Table 2 and in Figure 3. For example, the PC1 and PC2 values for the rat Acr gene and the homologous Drosophila Jon74E gene, described in the first row of Table 2, are –1.17 and –3.56, respectively.

Figure 3 presents the results of the correlation analysis between principal components PC1 and PC2 on the grounds of experimental data on pairs of homologous rat and Drosophila DEGs (Table 1). Each point in the figure corresponds to the PC values calculated for a pair of DEGs: Drosophila gene from FlyDEGdb and the homologous rat gene from RatDEGdb. The PC1 and PC2 values are plotted along the Y and X axes, respectively. We see that the red dash-dotted line PC1 = 0 divides all DEGs into two disjoint groups: (1) group of DEGs with PC1 < 0, indicating stress-induced downregulation in both rats and Drosophila, and (2) group with PC1 > 0, indicating stress-induced upregulation in both species, according to Equation (1).

We can see a qualitative difference between the two DEG groups (above and below the red line) found in our comparison of stress-induced changes in the expression of homologous Drosophila and rat genes. The DEG group with stress-induced downregulation (blue) demonstrates a highly significant ( p < 10–12) positive correlation between PC1 and PC2. By contrast, no correlation between PC1 and PC2 is observed in the second DEG group with stress-induced upregulation (green).

Unexpectedly, our results on the rat and Drosophila coincided with independent observations by D.Yu. Oshchepkov et al. (2025). They analyzed changes in the expression of homologous genes of the rat and human induced by stFress and hypertension, respectively. In both cases, a significant correlation between the first and second principal components was noted only in the stress-induced downregulation of homologous genes.

The correlation between PC1 and PC2 in the PC1 < 0 area, which corresponds to stress-induced downregulation in the human, rat, and Drosophila, implies that the species may share common molecular mechanisms for gene inhibition under stress conditions of different sorts.

The commonly known molecular mechanisms for gene expression downregulation under stress include the arrest of pre-mRNA splicing in eukaryotes (Yost, Lindquist, 1986; Cuesta et al., 2000) and translation inhibition (Bresson et al., 2020)

We used the STRING software (Szklarczyk et al., 2021) to assess the Gene Ontology (GO) term enrichment in the group of Drosophila genes with stress-induced downregulation (blue dots in Figure 3). The results are shown in Table 3.

**Table 3.**
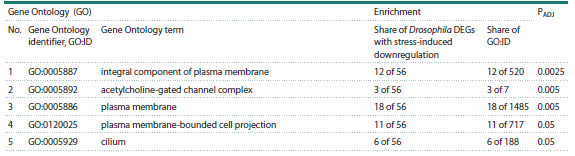
Assessments of gene ontology term enrichment in the group of Drosophila genes with stress-induced downregulation

The analysis revealed five GO terms in which the list of Drosophila genes with stress-induced downregulation is significantly ( p < 0.05) enriched. Four of the five (GO:0005887, GO:0005892, GO:0005886, GO:0120025, and GO:0005929) are directly related to components of the plasma membrane. The fifth term (GO:0005929, cilium), also belongs to this group, as cilia are specific organelles on the outer surface of eukaryotic cell membranes. This fact implies that the plasma membrane of Drosophila cells is one of the universal targets of stress factors described in FlyDEGdb. In this regard, note that stress-induced downregulation of Drosophila genes encoding components of plasma membranes in cells can slower their growth under stress. Our assumption agrees with the results presented in (Kassahn et al., 2009), where mechanisms of animal response to stress factors are reviewed. It should also be mentioned that M.F. Haque et al. (2025) detected an inhibition of Escherichia coli cell growth under stress.

To conclude, we note that the year 2023 marked the 80th anniversary of the famous maxim by Hans Selye “Stress is the spice of life” (Rochette et al., 2023). Our work once more illustrates the fundamental significance of the stress issue in life sciences.

## Conclusion

We developed the FlyDEGdb knowledge base, which is a body of experimental data on differentially expressed genes (DEGs) of Drosophila and their response to a broad range of stressing factors: cold, heat, dehydration, heavy metals, ionizing radiation, starvation, household chemicals, drugs, agricultural fertilizers, insecticides, pesticides, herbicides, and other toxicants. The knowledge base, storing information on the commonly used model species, D. melanogaster, can be employed by students of translational molecular biology and genetics of the human and animals, physiology, translational medicine, pharmacology, nutrition science, agricultural chemistry, radiation biology, toxicology, and bioinformatics

## Conflict of interest

The authors declare no conflict of interest.
